# Extended spectrum beta lactamase (ESBL)-producing *Escherichia coli* and *Klebsiella pneumoniae* in Indonesia and South East Asian countries: GLASS Data 2018

**DOI:** 10.3934/microbiol.2023013

**Published:** 2023-03-17

**Authors:** Sunarno Sunarno, Nelly Puspandari, Fitriana Fitriana, Uly Alfi Nikmah, Hasta Handayani Idrus, Novaria Sari Dewi Panjaitan

**Affiliations:** 1 Center for Research and Development of Biomedical and Basic Health Technology, Jakarta, Indonesia; 2 Center for Biomedical Research, Research Organization for Health, National Research and Innovation Agency (BRIN), Cibinong Science Center, Jl. Raya Bogor No. 490–Bogor Km. 46, West Java, Indonesia; 3 Center for Health Resilience and Resource Policy, Health Policy Agency, Jakarta, Indonesia

**Keywords:** antimicrobial resistance (AMR), ESBL, *Escherichia coli*, *Klebsiella pneumoniae*, bacterial infections

## Abstract

Antimicrobial resistance is the rising global health issue that should not be ignored. This problem needs to be addressed and professionally handled since it is starting to threaten global health, which eventually could lead to disaster. Extended spectrum beta lactamase (ESBL)-producing bacteria were found threatening lives, since most antibiotics were found to not be effective in treating patients with infections caused by those bacteria. ESBL-producing *Escherichia coli* and *Klebsiella pneumoniae* are the two most reported bacteria in causing the bacteremia and nosocomial infections worldwide. In this article, the prevalence of ESBL-producing *E. coli* and *K. pneumoniae* in causing blood stream and urinary tract infections in Indonesia were compared to the neighboring countries based on the global antimicrobial resistance surveillance system performed worldwide by World Health Organization (WHO). In this article, the prevalence of ESBL-producing *E. coli* and *K. pneumoniae* in Indonesia and its neighboring countries were assayed and compared in order to evaluate the antimicrobial resistances. By comparing the prevalence data to the neighboring countries, some insightful evidence and information was served to support improved health in Indonesia. Some hurdles and strategies in combating the antimicrobial resistances were further discussed. Eventually, an alternate solution to overcome the antimicrobial drug resistance should be well-provided, studied and implemented globally.

## 1. Introduction

Antimicrobial resistance (AMR), especially among Gram-negative bacteria, is a global health problem for current and future threats [1]. The worsening trend of AMR results in longer stays in hospitals, increased cost of health care which burdens both patients and governments, and higher mortality and morbidity. A previous study reported that the global mortality attributed to AMR was predicted to reach 10 million cases per year by 2050, which massively increases from the current estimated mortality of 700,000 cases per year globally [2]. With that prediction, the global political and socio-economic stability would be negatively impacted [3].

Extended spectrum beta lactamases are produced by specific bacteria called ESBL-producing bacteria which enable those bacterial cells to be more resistant to antibiotics and, therefore, their infections are harder to treat. A previous study performed by Matsumoto *et al*. tried to unravel the history of ESBL-producing *K. pneumoniae* and *E. coli* isolated from sputum obtained from patients diagnosed with respiratory tract infections [4]. The findings of their study mentioned the historical association that the intestinal colonization of ESBL-producing *K. pneumoniae* and *E. coli* causes the gastroesophageal reflex which leads to lower respiratory tract infection, while the bacterial migration from the anus to the meatus urinarius tract leads to urinary tract infection [4],[5]. The widespread presence of carbapenem and polymyxin resistance has been also reviewed in nations worldwide, which represents increased threat of infections resistant caused by resistant Enterobacteriaceae [6],[7]. Therefore, the roles of action of ESBL-producing *K. pneumoniae* and *E. coli* on health regarding various infectious diseases are getting serious. ESBL-producing bacteria, such as ESBL-producing *Escherichia coli* and *Klebsiella pneumoniae*, are reviewed and listed as the critical priorities for development of new antibiotics based on the data from World Health Organization (WHO) [8],[9]. WHO had adopted a global action plan (GAP) on AMR which consisted of five objectives, where developing the economic case for sustainable investment and increasing the investment value in new medicines, diagnostic tools, vaccines and other interventions are becoming part of the objectives [10]. Meanwhile, supporting the global action plans provided by WHO, the government of the United States has also designed and implemented the national action plans (NAP) in battling AMR since 2015, where One Health approach was highlighted in it [11]. Both NAP and GAP were designed as an effort to address urgent and serious drug-resistant threats, in this case, bacterial infections that could affect people around the world. Meanwhile, based on the NAP designed by the Ministry of Health in Indonesia, the importance of whole society engagement in every field to be aware of One Health approach in combating AMR nationwide is also emphasized [12]. The actions guided and performed according to both NAP and GAP in each country should be strengthened to reach the objectives planned since, recently, the transmission of multidrug resistant bacterial infection seems to be spreading more widely [13],[14].

## 2. Global antimicrobial resistance surveillance system (GLASS) and Tricycle Project

The World Health Organization (WHO) has responded to several activities, including the Global antimicrobial resistance surveillance system (GLASS) and the Tricycle Project [15],[16]. GLASS is focused on monitoring antimicrobial resistance against several types of bacteria, including *E. coli* and *K. pneumoniae*. Meanwhile, the Tricycle Project is focused on monitoring Extended Beta Lactamase-producing *E. coli* in human, animal (broilers) and the environment. In Indonesia, GLASS activities were begun to be executed in 2018 by collecting resistance data from nine hospitals across several provinces in the country. This data is important to see the pattern of antimicrobial resistances pattern in Indonesia, especially those happened in hospitals. Meanwhile, the Tricycle Project was carried out in 2017–2018 by involving two hospitals which were also GLASS sentinel sites. Therefore, a correlation between the two activities was predicted. In this article, the data of ESBL-producing *E. coli* and *K. pneumoniae* in Indonesia compared to neighboring countries based on GLASS data in 2018 obtained from WHO database was presented [17].

## 3. Blood stream infection and urinary tract infection caused by ESBL-producing *E. coli* and *K. pneumoniae*

ESBL-producing *E. coli* and *K. pneumoniae* are two infectious bacteria that have become a serious concern for nosocomial infections worldwide, especially Indonesia. *E. coli* is a common Gram-negative bacterium that belongs to the normal human microbiome. However, *E. coli* itself was also reported to be responsible for blood stream infections (BSI), which could be leading to community and hospital acquired BSI [18]–[20]. *K. pneumoniae* was well-known for being responsible pathogen for pneumonia, septicemia and urinary tract infections (UTI). In a previous reported study of ESBL-producing *K. pneumoniae* that were isolated from patients with nosocomial infections, showed high rates of resistances against ciprofloxacin (86.2%), tetracycline (80.9%) and nalidixic acid (78.7%) [21].

**Figure 1.**
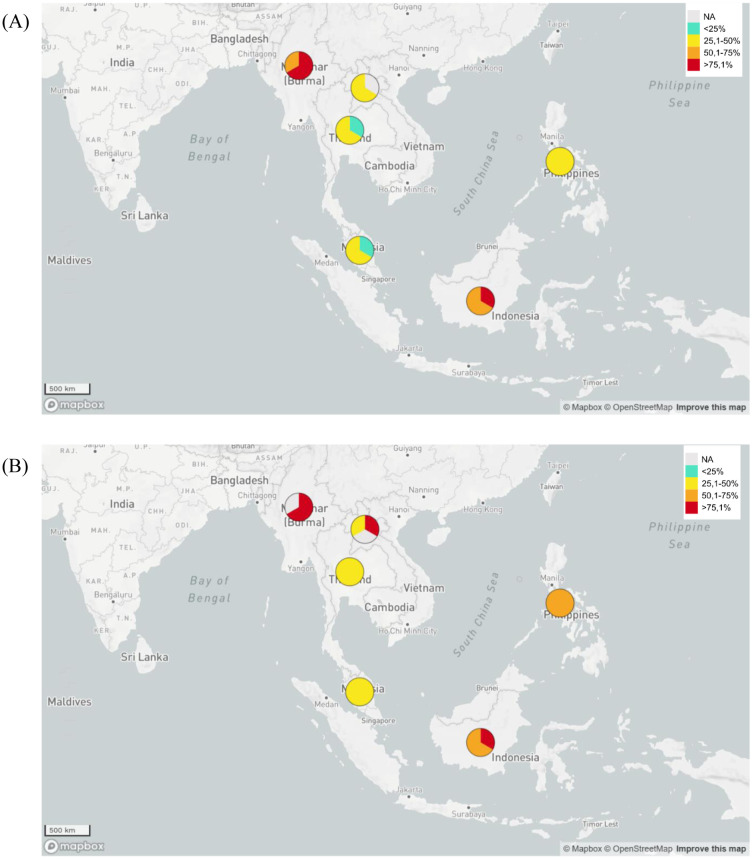
Map of the prevalence of ESBL-producing *E. coli* and *K. pneumoniae* causing bloodstream infections (BSI) in Indonesia and neighboring countries based on GLASS data in 2018. (A) Prevalence of ESBL-producing *E. coli* causing BSI. (B) Prevalence of ESBL-producing *K. pneumoniae* causing BSI.

**Figure 2.**
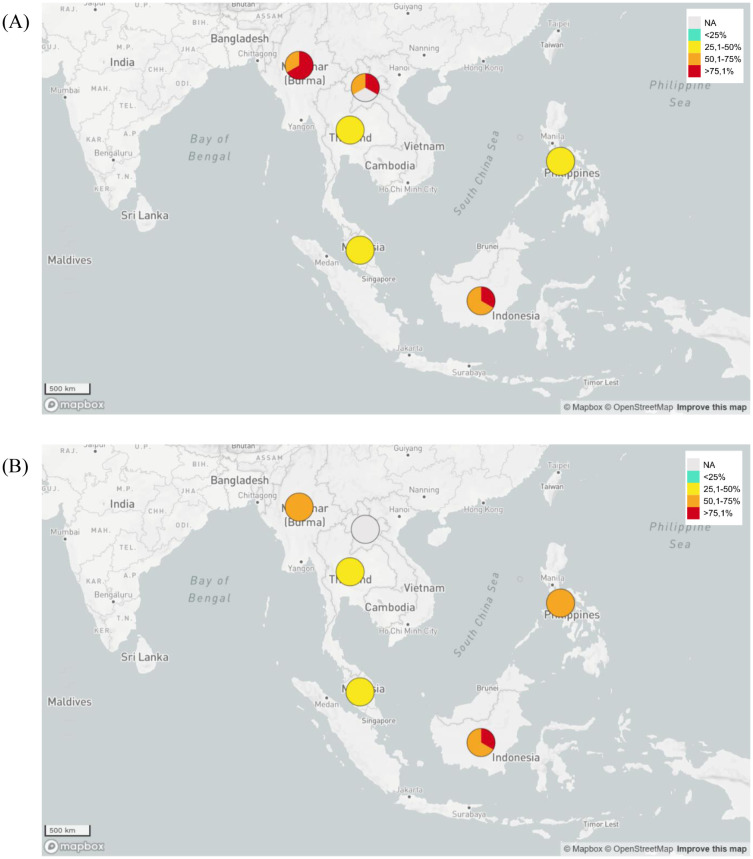
Map of the prevalence of ESBL-producing *E. coli* and *K. pneumoniae* causing urinary tract infections (UTI) in Indonesia and neighboring countries based on GLASS data in 2018. (A) Prevalence of ESBL-producing *E. coli* causing UTI. (B) Prevalence of ESBL-producing *K. pneumoniae* causing UTI.

Table 1 shows that ESBL-producing *E. coli* data on GLASS activities are similar to previous published Tricycle Project data [16]. In general, Indonesia has a high level of resistance to *E. coli* and *K. pneumoniae* to third generation cephalosporin antibiotics (ceftriaxon, ceftazidime and cefotaxime), similar to that possessed by Myanmar and Laos (Figure 1). Unfortunately, the prevalence data of ESBL-producing *K. pneumoniae* causing BSI with resistance toward ceftazidime treatment in Myanmar was not available. The prevalence data of ESBL-producing *K. pneumoniae* causing UTI with resistance towards those three antibacterial agents and ESBL-producing *K. pneumoniae* and *E. coli* with resistance towards cefotaxime were not available either. Based on this preliminary data, the government of Indonesia needs to evaluate and improve its AMR prevention program. Indonesia also needs to learn from neighboring countries with similar geographic and demographic conditions, but with lower level of prevalence of ESBL-producing *E. coli* and *K. pneumoniae*, such as Malaysia. Unfortunately, GLASS data from Singapore, Brunei and Timor Leste in 2018 based on the WHO report were not available. Among all listed countries whose data are available based on WHO report, Thailand had the lowest prevalence of ESBL-producing *E. coli* and *K. pneumoniae* causing BSI and UTI (Table 1 and Figures 1 and 2).

**Table 1.** Prevalence of ESBL-producing *E. coli* and *K. pneumoniae* causing bloodstream infection (BSI) and urinary tract infection (UTI) in Indonesia and neighboring countries based on GLASS data in 2018.

		Blood stream Infection (BSI)				Urinary Tract Infection (UTI)			
Countries		*K. pneumoniae*		*E. coli*		*K. pneumoniae*		*E. coli*	
		Mean	Range	Mean	Range	Mean	Range	Mean	Range
Indonesia	Ceftriaxon	73.5	68.0–78.3	72.3	67.0–76.7	68.0	64.0–71.5	64.7	62.0–66.9
	Ceftazidime	69.4	64.0–74.8	58.7	53.0–64.3	65.8	62.0–69.4	56.4	54.0–58.7
	Cefotaxime	80.0*	68.0–88.2*	87.3	78.0–93.2	82.1	67.0–91.0	80.0	71.0–87.0
Malaysia	Ceftriaxon	33.3	31.0–35.3	25.5	24.0–27.3	32.0	30.0–34.0	33.3	31.0–35.3
	Ceftazidime	33.1	32.0–34.3	18.5	18.0–19.5	32.3	31.0–33.6	33.1	32.0–34.3
	Cefotaxime	35.3	34.0–36.6	27.3	26.0–28.5	35.9	35.0–37.3	35.3	34.0–36.6
Phillippines	Ceftriaxon	55.1	52.0–57.8	33.8	31.0–36.6	56.6	54.0–58.8	41.2	40.0–42.6
	Ceftazidime	50.8	48.0–53.6	26.2	24.0–28.8	54.2	52.0–56.4	32.7	31.0–34.0
	Cefotaxime	57.6	54.0–61.1	40.7	37.0–44.7	61.5	59.0–64.5	46.0	44.0–47.9
Thailand	Ceftriaxon	27.9	24.0–32.3	36.1	33.0–39.0	47.4	44.0–51.0	41.3	39.0–43.2
	Ceftazidime	29.6	25.0–34.3	19.3	17.0–21.9	44.6	41.0–48.0	26.6	25.0–28.3
	Cefotaxime	27.7	24.0–32.2	36.8	34.0–39.9	47.4	44.0–50.7	41.9	40.0–43.7
Myanmar	Ceftriaxon	80.0	58.0–91.9	82.1	67.0–91.0	59.8	51.0–68.0	75.7	73.0–78.3
	Ceftazidime	NA	NA	75.0	53.0–88.8	54.1	43.0–64.9	65.0	62.0–68.3
	Cefotaxime	92.9	69.0–99.6	77.4	60.0–88.6	64.5	54.0–73.5	75.8	73.0–78.6
Laos	Ceftriaxon	30.6	18.0–46.9	45.9	37.0–55.2	NA	NA	75.9	66.0–83.6
	Ceftazidime	76.9	50.0–91.8	44.6	33.0–56.7	NA	NA	60.5	45.0–74.4
	Cefotaxime	NA	NA	NA	NA	NA	NA	NA	NA

NA: Not available

## 4. Hurdles to deal with ESBL-producing *E. coli* and *K. pneumoniae* resistance

In the middle of rapidly growing molecular microbiology knowledge and diagnostic techniques, in addition to the global research and development of novel antibacterial agents, the questions we should be asking now is why it takes so much effort and a long period of time to deal with AMR? Are we doing the right things? Could we possibly miss or neglect something, such as the deeper involved understanding of antibiotic resistance mechanism [5],[22]? Are we even closer to the “success” line regarding the AMR management? In addition, how can we predict that the global effort will eventually reach the goal in combating AMR worldwide? More and deeper studies are really required to give us at least a prediction to address and answer those questions.

The coliform-appearing ESBL-producing Enterobacteriaceae isolated in an urban West African rat population in a study performed by Schaufler *et al*. mostly consisted of ESBL-producing *E. coli* and *K. pneumoniae* with resistances to all type of antimicrobial drugs [23]. In a previous study performed by Yamasaki *et al*., the high prevalence of multiple-drug resistance was observed accompanied with the detection of ESBL genes, such as bla_CTX-M-1_, bla_CTX-M-2_, bla_CTX-M-9_, bla_CTX-M-15_, bla_SHV_ and bla_TEM_, which were highly detected in ESBL-producing *K. pneumoniae* in Indonesia [21]. Certain additional bacterial genes, such as *aac(3)-IId-like*, *strA*, *strB*, *aac(3)-IIa-like*, *strA-like*, *strB* and *aac(6′)Ib-cr*, responsible for ESBL production and multi drug resistances were figured out [23]. The non-stoppable distribution of these bacterial genes is the absolute reason underlying the hurdles to handle the AMR cases globally [24].

## 5. Future strategies in combating antimicrobial resistances

Several approaches, such as increasing the global collaboration between governments non-government organizations (NGOs), professional groups and international agencies have been addressed as the approaches to combat antimicrobial resistance, in addition to rational drug use, infection control and prevention, antimicrobial surveillance, ban on OTC antibiotics, giving proper education and motivation, increasing and elaborating the research and development of drug and vaccine, new AMR programs, global policies formulation and building a global AMR committee [25]. Moreover, implementation of the global action plan on AMR planned by WHO, followed by enactment of the GLASS and completion of National Action Plan, could seriously be a comprehensive approach in order to combat the global health issue of antimicrobial resistance [26]. However, taken from the point of view of molecular microbiology, there are many reports highlighted and proposed novel ideas in order to handle AMR, such as phage therapy, antimicrobial peptides, nanoparticles [27], essential oils [28],[29], antisense peptide antimicrobial therapeutics [30], transplant using isolated fecal microbiota [31],[32] and the formation of new antibiotic association, which were listed and well-reviewed by Mdarhri *et al*. [8]. Moreover, the approaches from pharmacokinetic and drug designs in developing the antibiotics drug delivery systems using novel potential organic compounds, such as liposomes, lipid-based nanoparticles, polymeric micelles, polymeric nanoparticles and drug delivery utilizing inorganic compounds, such as silver, silica, magnetic, zinc oxide (ZnO), cobalt, selenium and cadmium, have been reviewed elsewhere [27]. From those proposed novel strategies, none of them declare success since more studies are still being performed and needed. Hopefully, the global health problem regarding AMR could be overcome in the near future. The high cost of treating and handling the cases of AMR could affect the economy of a country. Therefore, wise investment in economic factors and uncertain returns on investment on new antibiotic or drug development may not be the only answer for this issue [33]. In summary, the global guidance and national action plans designed by each government should be periodically evaluated, the data should be analyzed and well-announced to raise the awareness towards AMR, and the efforts in One Health approach should be strengthened and well-mediated to combat this global health threat.
